# A Case of Urticaria, Induced by the Ingestion of the Allium Porrum

**Published:** 1857-10

**Authors:** William G. Meachem

**Affiliations:** Warsaw, N. Y.


### ART. V.—*A Case of Urticaria, induced by the Ingestion of the Allium Porrum.* By WM. G. MEACHEM, Warsaw, N. Y.

Sometime in April, I was summoned to the bedside of Master Atwell, aged — years. I found him in the height of a severe paroxysm of dyspeptic colic, the stomach, equally with the bowels, participating in the spasm. Upon an inquiry into the probable cause of the attack, I learned that, upon the day previous to the first appearance of the distressing symptoms under which he was then laboring, my patient, in company with one or more other boys, had eaten freely of the raw leek, which they had found in their rambles through the wood. At once linking together these two circumstances, the ingestion of the vegetable and the occurrence of the colic, as cause and effect, I addressed to the case such remedies as reason and authority dictated. A mercurial cathartic was forthwith administered, calomel being allowed the preference in consideration of the excessive gastric irritability present. An emetic was proscribed, since I had reason to believe that the stomach had already been emptied of its contents. Anodynes were ordered for subsequent employment. The abdomen was fomented with *infusio lupuli humuli.*

Upon visiting him the succeeding day, I perceived that he was nearly covered with the wheals of *urticaria conferta*, the principal seats of the eruption being the loins and the internal aspects of the fore-arms. The characteristic pruritus was unusually developed. In this instance the face, an unusual locality, was also well spoted.

I have been induced to give publicity to this case by the circumstance of never having seen, in standard works upon theory and practice, any specific mention of this variety of esculent as a producing cause of nettle-rash. Quite extended lists of articles of diet capable of evolving this peculiar exanthem are given by various authors, but not one of them, so far as my reading extends, individualizes the *allium porrum.* The frequent occurrence of this tuber in our wooded districts, and the well-known inclination of childhood to freight the alimentary canal with any and every thing that is sapid and masticable, render it highly probable that leeks are much oftener eaten and much oftener occasion attacks of colic and, it may chance be, urticaria, than has heretofore been supposed. In truth, it was but a few days subsequent to the occurrences just narrated, that I prescribed for a married lady who had also made herself a victim of the contortions of colic through identically the same means, the ingestion of leeks, urticaria however, did not ensue in this instance.

What is the essential pathological condition of the exanthem of urticaria? has ever been a query of interest to me, and I here beg the indulgence of the reader while I assay, in a very superficial manner, an answer thereto. In those cases of the disease in which the cause is manifest, for instance, such as are induced by certain articles of diet (and by far the larger number are originated in this way) we have a foundation laid upon which we may build explanatory structures with firm reliance upon their truthful permanence in which we are unable to identify the causative influence, I doubt not that it is the same, or at least, similar, though less readily recognized.

In attacks due to particular ingesta, the cutaneous efflorescence may be accounted for in the following three several ways:

1st. It may be ascribed to the propagation of continuous sympathy from the irritated and inflamed mucous lining of the stomach and intestines.

2dly. The exanthem may essentially consist in active congestion of the sudoriparous and, perchance, the sebaceous glandulæ and the adjacent parts, occasioned by the irritating presence of various constituent principles of the ingesta of which the body endeavors to free itself through the medium of the cutaneous exhalent system.

3dly. The glandular congestion may result from the acridity of new compounds originated within the body, probably in the circulating current, either out of the primitive elements of the same ingesta, or from chemical reäction upon materials already formed, or simply in consequence of the disturbance in function which their presence provokes, which new compounds also seek an outlet through the skin. It may be well here to bear in mind that the the intestinal glands, as well as the cutaneous, assist in the elimination of the various materies morbi, and I doubt not that the mucous membrane of the alimentary canal participates, in an equal degree, in the efflorescence, modified somewhat, it may be, by its location. Indeed, authors state that the eruption has been seen in the cavity of the mouth.

I will now briefly consider each of the foregoing hypotheses; and first, the sympathetic influence. It is highly probable that urticaria, in some cases, is a sympathetic manifestation propagated from an irritated or inflamed gastric or intestinal mucous membrane; and this supposition appears the more reasonable from the fact that those articles of food which are the producing agents of the attacks, are usually of a very indigestible character, peculiarly liable to irritate the primæ viæ. The mere mention of some of these substances confirm the truth of the statement. Among them may be named raspberries, strawberries, salted and smoked meats, green cucumbers, &c. The various kinds of shell-fish, particularly muscles, which are so commonly productive agencies, doubtless owe their energy as such to some idiosyncracy of the patient, rendering what is ordinarily inoffensive, acrid, and irritant.

As it is needless to delay with the proof of a doctrine so well established as that of continuous sympathy, and as the great similarity, closely approximating identity, of structure in mucous and tegumentary tissues, and their continuousness, are truths susceptible of physiological demonstration, I may dismiss the examination of this first assumed cause with simply adducing a few instances in which this sympathy cannot but be apparent. In intestinal irritation from worms, the labial, nasal, guttural, vaginal, urethral, and anal mucous membranes sympathize, and itching of those parts becomes a prominent symptom, inducing, when located in the throat, a peculiar dry cough denominated verminous. When cough appears the bronchial lining may also possibly sympathize. A purulent discharge from the vagina also sometimes arises in this way.

Again, when the pulmonary membrane is irritated and inflamed, as in catarrh, influenza, bronchitis, &c., the gastric sympathies and indigestion ensues. The same consequences follow in the wake of the various forms of cynanche. In dentition, also, the intestinal lining is sympathetically affected and diarrhœa results. In fact dentition and acidity of stomach are observed causations of urticaria. Now, in the last cases the exanthem is manifestly sympathetically called into being. Why may it not occasionally be similarly caused in other cases? It is true, that in some instances there may be an actual *extension* of the affection from the stomach and bowels to the skin, but in a larger number it is, doubtless sympathetically propagated. The institution of this cutaneous eruption is, indeed, one of the conservative efforts in which nature delights. It is nature's *revulsion:* the external irritation derives from the internal. And herein is seen the wisdom of the substitution, that, whereas the original irritation is in one of the peculiarly vital organs, the secondary is in an organ the interruption of whose function is less immediately hazardous, since its duty can be vicariously executed by the lungs, the kidneys and the liver. It is a cardinal indication, therefore, to encourage, rather than obstruct, the evolution of the cutaneous exanthem.

Let me now turn to the second hypothesis. The arguments adducible in support of this theory, are chiefly to be derived from the physiological character of the skin and from analogy. Physiology asserts that the sudoriparous and possibly the sebacious glandulæ are conduits of exit for not only the normal waste of the system, but also for such constituents of aliment as are absorbable, but yet unassimilable, or not required by the body. Hence the conjecture is far from unreasonable that materials of food which excites the disease, may reach the tegumentary glandulæ in their outward passage, and therein, by their irritating physical or chemical properties, institute congestive action or rather *in*äction. These materials may be either the saline matters of the food, whose crystals, by reason of their conformation, are peculiarly fitted for mechanical irritation, or acids, both of which are unusually abundant in vegetables, the variety of ingesta most prone to establish the efflorescence. Possibly lactic acid, the normal excretion of the sudoriparous glandulæ, may, in some instances, by augmented quantity or strength, become an excitant of congestion.

I have hitherto denominated the cutaneous eruption active congestion, rather than inflammation; because its characteristics indicate the former much more certainly than the latter. Its usually transient appearance, its sudden retrocession and return, the invariable absence of suppuration, all discountenance the idea of inflammation, but favor that of congestion. The pathology of the distinctive wheals seems to be this: The acrid materials withdrawn from the blood by the secreting action of the glandulæ, mechanically obstruct the calibres of a limited number of adjacent perspiratory ducts, this clogging being aided by the compressing force exerted upon the ducts from without by the increased afflux of blood into the encompassing plexus of vessels. The glandulæ consequently become distended, and hence arise the elevations, denomenated wheals. The whitish color of the surface of these protruberances may be due to the pressure made by the distended glandulæ upon the superjacent blood-vessels, partially obliterating their cavities and thereby diminishing the amount of red fluid circulating in them. The generally diffused erythema, out of which, as a bed, the wheals arise, is probably an active congestion of the skin, unaccompanied by obstruction of the perspiratory ducts. This explanation also harmonizes with the sudden disappearance and reäppearance of the erythematous blush and the wheals. As soon as the obstructions in the ducts are removed, the tumor must necessarily abate, since there are no deposits of lymph, pus, blood, &c., to be gotten rid of by absorption or otherwise. And equally so with the erythema: When the *vis a tergo* which impels, or the *vis a fronte* which solicits, an active determination of blood, subsides, almost simultaneously will the congestion cease. The peculiar hardness of the tumors might induce the belief that those tumors were depositions of lymph, but the more philosophical reference of that hardness is to distention, which is well known to be attended with this physical quality. A bladder filled to repletion with fluid, presents an apt illustration. If this eruption ever is inflammatory, the inflammation must be similar to, or identical with, that variety known as erysipelatous, the erratic nature of which is its distinguishing characteristic.

The well-observed fact that more or less serious constitutional disturbance invariably ensues upon the retrocession of the efflorescence, corroborates the opinion that that efflorescence is induced by the elimination of morbific material from the blood, any interruption of which elimination necessarily occasions the retention of that material in the system, and resultant constitutional irritation and fever. Hence, also, with this view of the disease, a perfect development of the exanthem should be sedulously encouraged.

The chief argument derivable from analogy in advocacy of this second hypothesis, is the fact that certain odorous principles of alimentary and medicinal substances are frequently detectable in the pulmonary and cutaneous exhalations, thus demonstrating that certain materials can enter the stomach, pass through the entire round of the circulation, and at last, unchanged, make their exit through one or more of the emunctories, The odors of onions, garlic, the terebinthinates, &c., are cases in illustration. Is it not supposable that other less odorous, also less perceptible, may with equal facility accomplish the same circuit.

Again, the profuse evacuations from the skin, intestines, &c., which so often occur coincidently with the period of amendment in idiopathic fevers, and which are designated, critical discharges, strongly confirm the pathological idea that this class of diseases owe their existence to the presence in the blood of a peculiar virus, which is removed from the system by these copious perspirations and diarrhœas. May not nettle-rash have a similar origin and remedy? May not a morbific material be its cause and the cutaneous eruption, its critical discharge, or rather the effect of its critical discharge?

Let me, in the last place, cursorily examine the third hypothesis, in support of which all, or nearly all, that has been advanced in behalf of the second proposition, may, with equal truth, be predicated. It is from the domain of analogy, however, that we derive our strongest evidences in maintenance of this last theory. The major exanthemata, barring their contagiousness, present many features in common with urticaria, not the least noticeable of which is the efflorescence. Now, these diseases, it is almost universally conceded, are due to the presence in the nutritive current of certain specific poisons, which in their passage through the depurating glandulæ of the skin, excite, in some instances, as variola, inflammation of the tegumeetary tissue; in others, as rubeola and scarlatina, active congestion. Is it not as probable that in at least some cases of urticaria, though not a specific virus, a definite materies morbi may be the productive agent of their peculiar exanthem?

Indeed, there is nothing unreasonable in the supposition that each one of these three alleged influences may, at times, separately prove a causation of urticaria, though, in perhaps a greater number of instances, a combination of two or more is requisite to the origination of the malady. This view of the subject may also afford a solution of the cause of the great diversity in febrile intenseness observed in different attacks of nettle-rash. In some, it is well known, there is little or no febrile excitement; in others it is alarmingly developed. Now, may we not conjecture that, when the febrile movement is slight or does not exist, the mildest of these three causes, sympathetic action, singly operates, and that, when the fever is more considerable, one or more of the intenser influences, or a combination of all, is called into exercise?

